# Donor-recipient specificity and age-dependency in fecal microbiota therapy and probiotic resolution of gastrointestinal symptoms

**DOI:** 10.1038/s41522-023-00421-4

**Published:** 2023-08-03

**Authors:** Qinglong Wu, Prapaporn Boonma, Shyam Badu, Nazli Yalcinkaya, Sik Yu So, Kevin W. Garey, Kent Williams, L. Eugene Arnold, Robert J. Shulman, Richard Kellermayer, Tor C. Savidge

**Affiliations:** 1 Department of Pathology & Immunology, Baylor College of Medicine, Houston, TX USA; 2 Texas Children’s Microbiome Center, Department of Pathology, Texas Children’s Hospital, Houston, TX USA; 3 Faculty of Medicine, King Mongkut’s Institute of Technology Ladkrabang, Bangkok, Thailand; 4 Department of Pharmacy Practice and Translational Research, College of Pharmacy, University of Houston, Houston, TX USA; 5 Department of Pediatrics, Ohio State University & Nationwide Children’s Hospital, Columbus, OH USA; 6 Department of Psychiatry and Behavioral Health, Ohio State University, Columbus, OH USA; 7 Department of Pediatrics, Baylor College of Medicine & Texas Children’s Hospital, Houston, TX USA

**Keywords:** Health care, Microbiome

## Abstract

Fecal microbiota transplantation (FMT) has proven to be an effective treatment for recurrent *Clostridioides difficile* infection (rCDI) in both adult and pediatric patients. However, as microbiome development is a critical factor in children, it remains unclear whether adult fecal donors can provide age-appropriate functional restoration in pediatric patients. To address this issue, we conducted an integrated systems approach and found that concordant donor strain engraftment, along with metabolite restoration, are associated with FMT outcomes in both adult and pediatric rCDI patients. Although functional restoration after FMT is not strain-specific, specialized metabolic functions are retained in pediatric patients when adult fecal donors are used. Furthermore, we demonstrated broad utility of high-resolution variant-calling by linking probiotic-strain engraftment with improved gastrointestinal symptoms in adults with irritable bowel syndrome and in children with autism spectrum disorder. Our findings emphasize the importance of strain-level identification when assessing the efficacy of probiotics and microbiota-based therapeutics.

## Introduction

*Clostridioides difficile* infection (CDI) is the most common healthcare associated infection in the USA and is a US Centers for Disease Control and Prevention threat level urgent pathogen^1,2^. The antibiotic vancomycin is most commonly used for the primary treatment of CDI^3,4^, but its broad-spectrum properties exacerbate gut dysbiosis, rendering patients vulnerable to disease recurrence^4^. Additionally, antibiotics that broadly disrupt the gut microbiota can lead to colonization and subsequent co-infection by other significant multidrug-resistant pathogens, such as vancomycin-resistant *Enterococcus*^5^. Therefore, the restoration and maintenance of gut microbiota is critical in the clinical management of CDI patients with infectious disease. It is noteworthy that fecal microbiota transplantation (FMT) is a highly effective treatment in preventing recurrent (r)CDI in ~80–90% of patients^6,7^.

The characterization of gut microbiota restoration is crucial for comprehending clinical responses to FMT. Prior studies have primarily relied on 16S rRNA-based analyses, using alpha-diversity indices, Bray-Curtis dissimilarity indices, and taxonomic abundance profiles. Those were generated from 97% similarity-based operational taxonomical unit (OTU) tables to monitor donor microbiota engraftment post-FMT^8–10^. However, these methods do not accurately define engraftment signatures, as they cannot identify donor-specific bacterial strains. Due to the ability of whole-genome sequencing (WGS) to detect and assemble genomes at the strain-level, shotgun metagenomic data is a powerful approach to analyzing donor-specific engraftment following FMT^11–14^. An alternative tool for identifying different strains of the same species from 16S amplicon data which is not possible using 97% similarity-based operational taxonomical unit (OTU), is the amplicon sequence variant analysis (ASV)^15^. However, ASV has not yet been utilized in tracking fecal donor strains in FMT cohorts, a method that we comprehensively benchmark and adopt in the present work.

While most FMT trials have concentrated on adult rCDI, fewer studies have investigated clinical efficacy or microbiota restoration in pediatric rCDI. As the gut microbiota is still developing in children^16^, it is crucial to consider developmental-specific aspects of gut microbiota restoration in pediatric patients when FMT is performed using healthy adult fecal donors. In this study, we conducted a comprehensive multi-omics investigation to identify critical features that associate with clinical FMT outcomes in pediatric rCDI, providing in-depth microbiome and functional analyses that were validated in adult FMT cohorts. We also evaluated the utility of strain-level resolution as determined by ASV to assess the clinical efficacy of probiotic engraftment in adults with irritable bowel syndrome and in children with autistic spectrum disorder who suffered from gastrointestinal symptoms.

## Results

### Microbiome variability in adult FMT donors

**Fig. 1:**
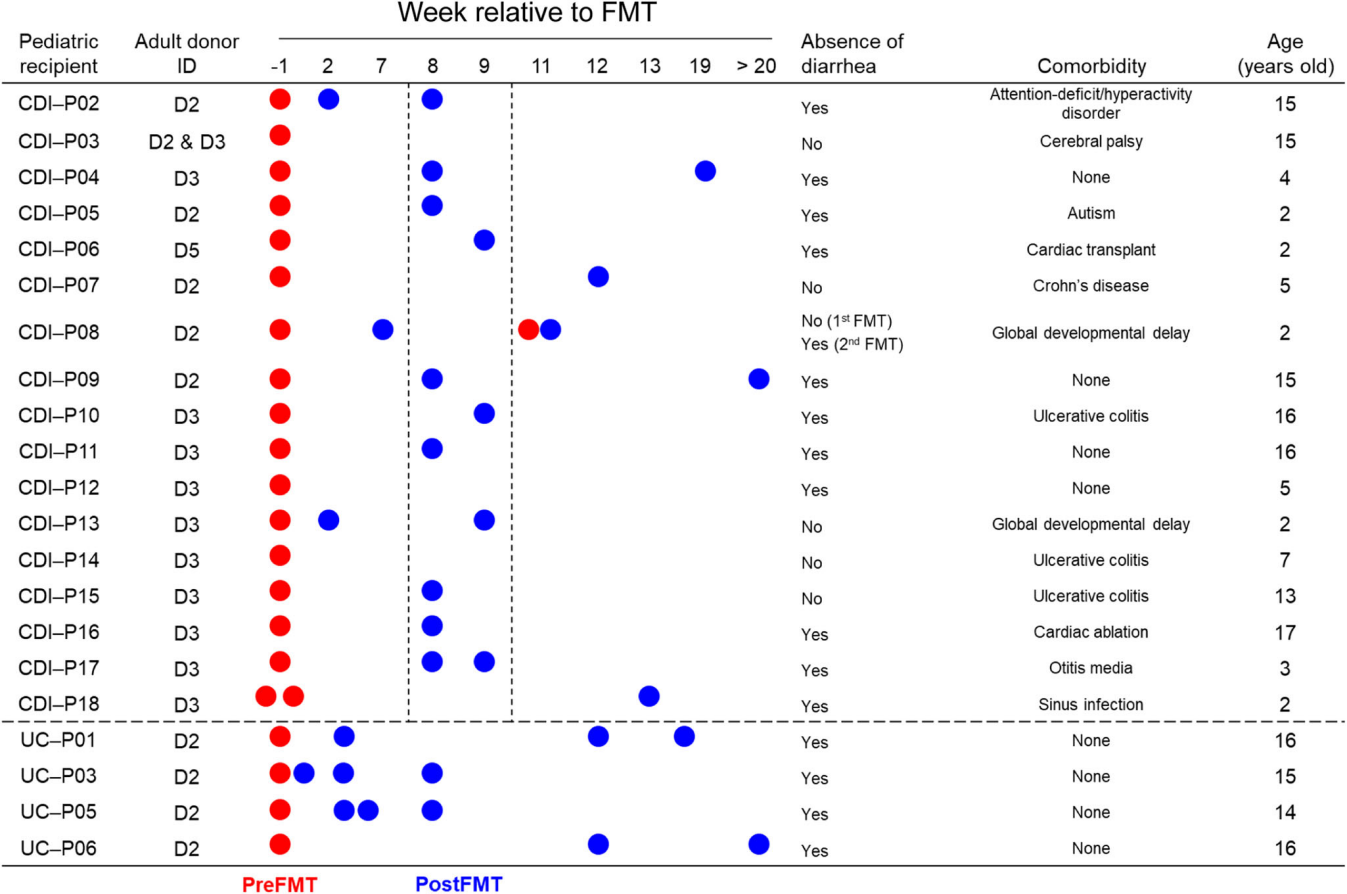
Pediatric recurrent CDI and ulcerative colitis patients receiving FMT from adult donors. Eighteen pediatric patients presenting with rCDI were initially administered antibiotics, specifically vancomycin, to reduce *C. difficile* vegetative cells in the colon. This was followed by colonoscopy-delivered FMT utilizing healthy microbiota from adult donors for reconstitution in the pediatric patients. The primary outcome measure was the resolution of diarrhea after 8 weeks for patients with rCDI, while diarrhea-free status during FMT served as the primary outcome for patients with ulcerative colitis (UC).

**Fig. 2:**
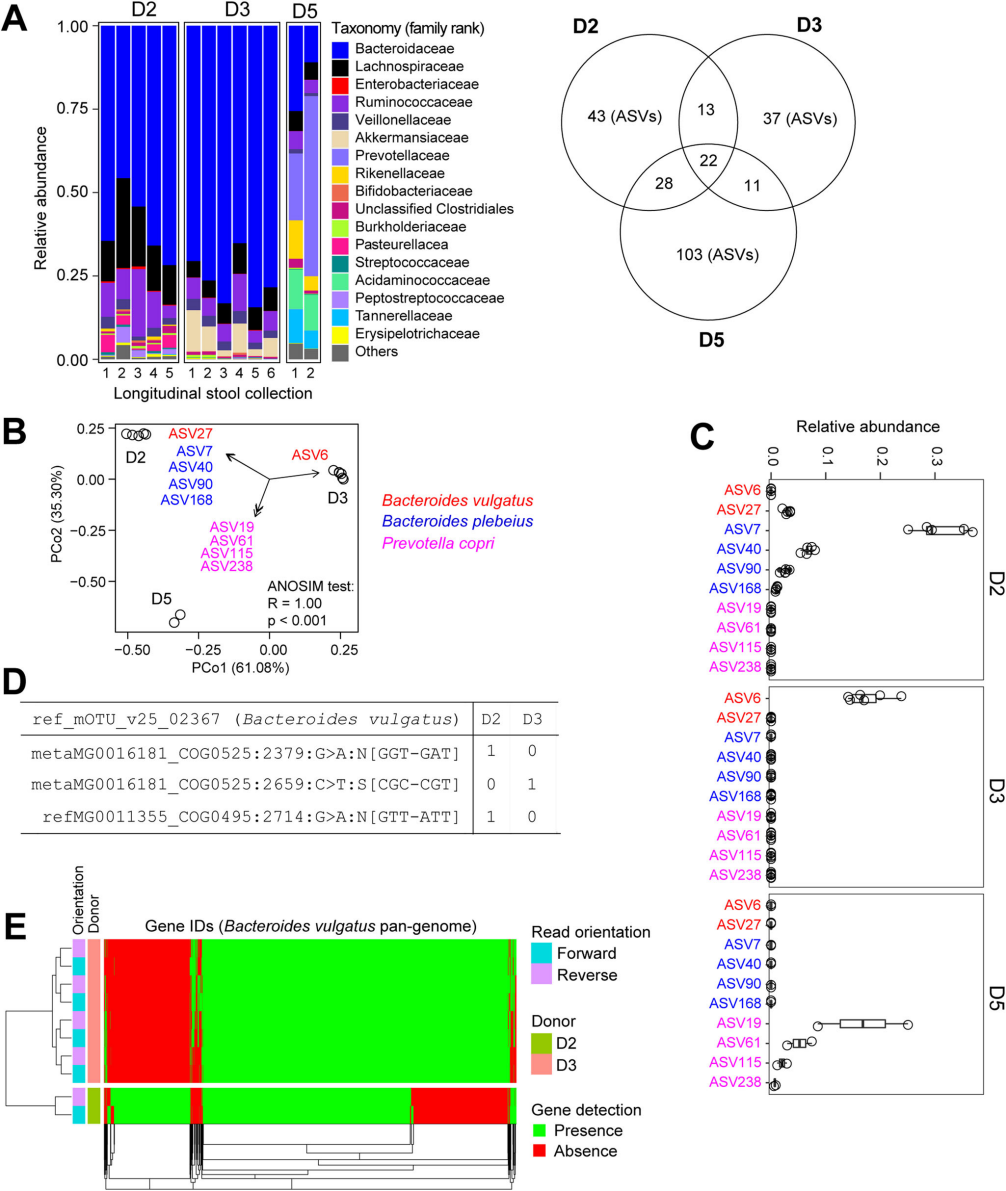
Single nucleotide variant profiling differentiates FMT donors. **A** Taxonomic abundance at family rank for three fecal donors showing stability of dominant *Bacteroides* (D2 & D3) and *Prevotella* (D5) over 7 months. Venn diagram shows the shared and non-shared ASVs among three donors. The presence of an ASV in the donor was determined by its detection in at least 50% of all longitudinal stool samples collected from that donor. **B** Amplicon analysis with single nucleotide resolution (ASVs) differentiate individual donors. *β*-Diversity analysis was performed with principal coordinates analysis using abundance-weighted Jaccard metric distance profiles. The envfit function in the vegan package was used to identify significant donor-specific ASVs (filter parameters: r2 > 0.8; Benjamini–Hochberg adjusted *p*-value < 0.02). Only ASVs with the same species call (BLCA annotation score cut-off of 85 using 98% identity and 98% coverage for alignment) are illustrated. **C** Relative abundance of ASVs that consistently differentiate individual donors. **D** Representative allele frequency of two single-copy marker genes of *Bacteroides vulgatus* by mOTUs SNV profiling of WGS data confirmed that ASVs represent distinct strains in donors D2 and D3. **E** PanPhIAN3 detection profiling of *Bacteroides vulgatus* pan-genomes in WGS data stably demonstrate distinct gene content of donor-specific *Bacteroides vulgatus* strains in donors D2 and D3.

Assessing microbiome variability and stability in fecal material used for FMT from healthy donors is a crucial step in identifying the reconstitution of the donor’s microbiome in the recipients, particularly in the case of multiple FMT procedures. In a previous FMT study, we treated pediatric rCDI cases over a wide age range (2–18 years) using three adult fecal donors for colonoscopy-delivered transfer^17^. Two universal donors, D2 and D3, were used to treat 21 children with rCDI (*n* = 17) or ulcerative colitis (UC; *n* = 4), and one self-designated donor, D5, was used to treat a single familial recipient (Fig. 1). Metagenomic sequencing and compositional analysis of longitudinal donor specimens revealed that universal donors D2 and D3 were stably dominated by *Bacteroides*, while D5 was *Prevotella*-enriched (Fig. 2A). These compositional differences were confirmed by distance-based unconstrained ordination analysis at the ASV level, showing distinct clustering among the three donors (Fig. 2B). By contrast, intra-individual specimens collected over a minimum of 7-months clustered tightly for each donor (Fig. 2A, B), demonstrating stability in microbiome composition for two universal donors who’s preparations were repeatedly used to treat patients with UC and rCDI in our studies.

Although donors D2 and D3 were both *Bacteroides*-enriched, specific dominant ASVs differentiated these specimens e.g. ASVs 7, 40 and 168 annotated as *Bacteroides plebeius* were only present in donor D2 (Fig. 2C). Donor-specific compositional differences extended to the strain-level, for example, ASV6 and ASV27 annotated *Bacteroides vulgatus* after BLASTn search with 100% sequence alignment coverage and identity (Fig. 2B). Single nucleotide variation (SNV) mapping of metagenomic reads to 10 universal marker genes in the mOTUs database confirmed the presence of different sequence variants of *B. vulgatus* in donors D2 and D3 (Fig. 2D). PanPhlAn3 analysis of metagenomic data using pan-genomes of *B. vulgatus* for read mapping further validated the different gene content of *B. vulgatus* in donors D2 and D3 (Fig. 2E), supporting the stable presence of strain variants in the two donors. Stable strain differences between donors D2 and D3 were also confirmed using the SameStr pipeline^18^ (Supplementary Data). By analyzing ASVs and metagenomic sequences, we found donor-specific microbiome signatures at the species- and strain-rank, which helped us to characterize donor-derived engraftment in multiple FMT recipients with different comorbidities and over a significant developmental age range (toddler to adolescent).

### Sequence variant profiling identifies donor-specific microbiome engraftment in FMT recipients

**Fig. 3:**
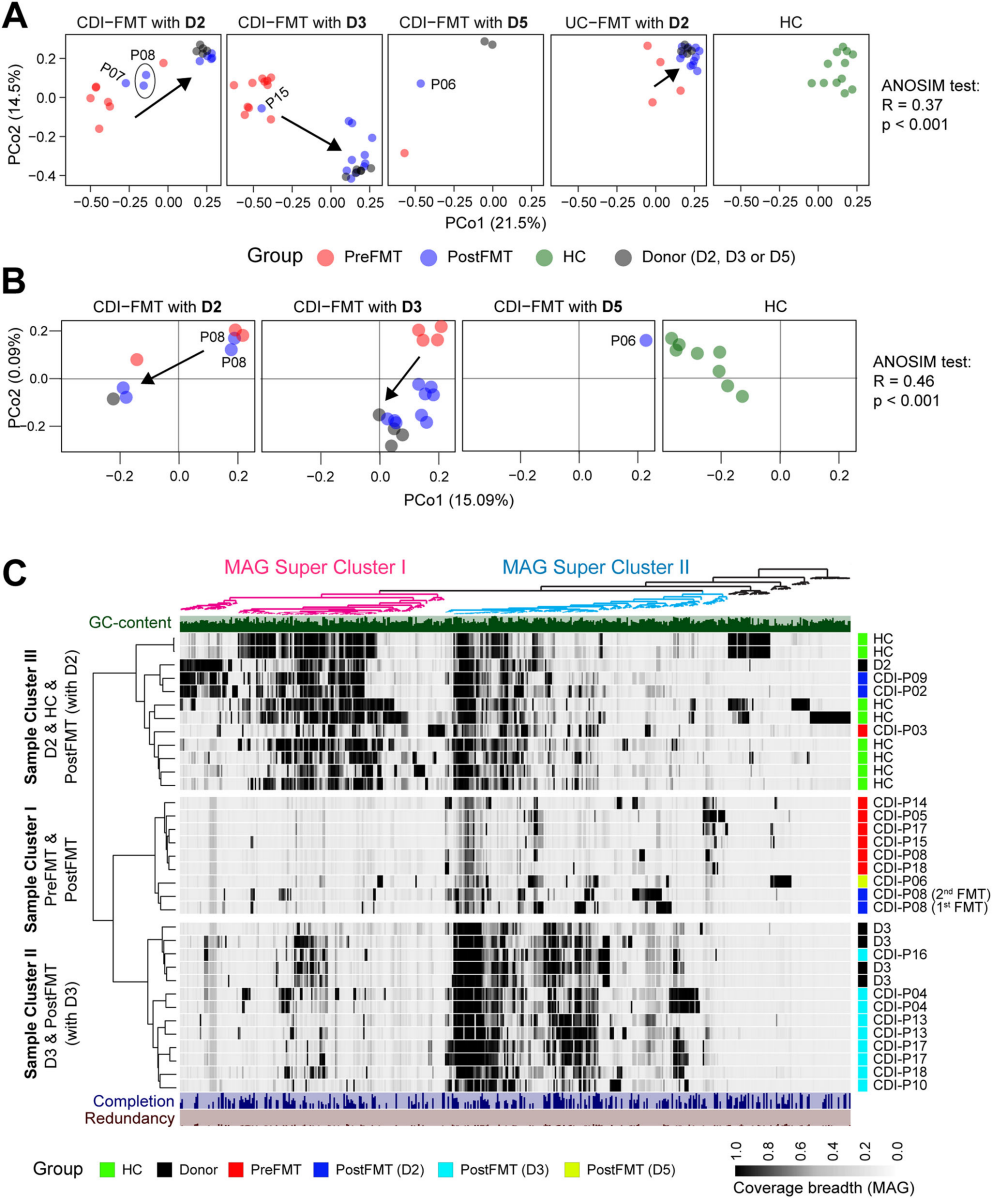
Donor-specific microbiota engraftment in rCDI patients receiving FMT. **A** Donor-specific microbiota engraftment in rCDI recipients is evident in a *β*-diversity analysis using principal coordinates generated with abundance-weighted Jaccard distance profiles. **B** *β*-Diversity analysis of mOTUs 10 marker genes-based SNV profiling of shotgun metagenomic data. Binary Jaccard distance profiles were calculated using mOTUs SNV profiles. **C** Donor-specific microbiome engraftment was validated by metagenomic assembly of genomes (MAGs) using the Anvi’o platform. Detection profiles (view: mean_coverage_Q2Q3) of donor-specific MAG bins are illustrated by hierarchical clustering analysis. Shotgun metagenomic data for patients P07 and P15 is not available due to insufficient yield of high-quality DNA.

Since each study subject received FMT from a single universal donor, this enabled us to track engraftment of donor-specific taxa in different disease recipients. Whereas taxonomic abundance profiling of ASVs showed a high degree of similarity between post-FMT and donor fecal samples (Supplementary Fig. 1), taxonomic redundancy above species-rank prevented us from assigning donor-specific engraftment with accuracy. However, by stratifying donor-recipient pairings using beta-diversity analysis of ASV profiles, we were able to illustrate donor-specific engraftment (Fig. 3A), a finding that was confirmed by the engraftment of top ranked donor-specific ASVs in FMT recipients receiving corresponding donor materials (Fig. 2B, C and Supplementary Fig. 2). By tracking all donor ASVs in each FMT recipient we demonstrated that a large proportion of these donor ASVs engrafted in recipients by replacing pre-FMT dysbiotic consortia (Supplementary Fig. 3).

Notably, sample clustering varied considerably depending on the unconstrained ordination method used (PCoA vs NMDS), with common distance metrics showing profound differences (Supplementary Fig. 4). For example, four beta-diversity metrics showed donor-specific microbiota engraftment in rCDI recipients, whereas two popular methods – PCoA with weighted UniFrac distance and NMDS with Bray-Curtis distance failed to recapitulate this finding. In order to eliminate the possibility of spurious ASVs (not chimeras) contributing to this disparity, we implemented a stringent abundance filter threshold of 0.01% (resulting in a reduction of ASVs from 1039 to 409). Even after this filter was applied, we still observed donor-specific engraftment (Supplementary Fig. 4). To gain further insights into the inconsistent findings, we conducted a comprehensive evaluation of these beta-diversity ordination methods. The following observations were made: (1) Jaccard and unweighted UniFrac metrics prioritize the analysis of rare features, while Bray–Curtis and weighted UniFrac matrices highlight abundant features among samples. (2) Unlike NMDS, which focuses on maximizing rank-order correlation, PCoA aims to establish concordance between the ordination and the original space, thereby facilitating enhanced sample discrimination. Overall, the abundance-weighted Jaccard metric, which takes into account both abundance and detection data for distance calculation, emerged as the most effective approach for identifying donor-specific ASV engraftment in patients with rCDI. Consequently, this metric was employed for further analysis of donor engraftment (Fig. 3A).

To validate our approach of using ASV profiling to identify donor-specific microbiome engraftment in FMT recipients, we analyzed shotgun metagenomic sequences generated from identical DNA preparations. Surprisingly, using species profiles generated by MetaPhlAn3, mOTUs and Kraken2 we failed to demonstrate similar donor-specific engraftment by abundance-weighted Jaccard distance metric (Supplementary Fig. 5). This deviation from the ASV profiling could be due to taxonomic redundancy at species level since these methods do not differentiate strain differences across samples. To further explore this possibility, we used taxonomy-agnostic SNVs profiling. Metagenomic allele frequency profiles were generated using the mOTUs pipeline with its pre-built 10 universal bacterial single-copy reference genes. Using PCoA-based unconstrained ordination with binary Jaccard distance metric for SNV profiling, we confirmed the ASV findings by demonstrating donor-specific microbiome engraftment, i.e., donor samples significantly clustered with paired Post-FMT compared with Pre-FMT specimens (Fig. 3B). We validated our metagenomics approach by independently demonstrating donor-specific microbiome engraftment in two previously reported FMT cohorts^11,12^ (Supplementary Fig. 6), supporting the use of strain-level detection to determine how donor engraftment relates to clinical FMT efficacy.

Because metagenomic SNVs profiling uses a reference-based strategy^11,12,18^, a potential limitation of this approach is that uncultured species are not included in the analysis. To assess this limitation, we performed taxonomy-agnostic, reference-free, de novo co-assembly and binning of metagenomic data using the Anvi’o platform^19^. Hierarchical clustering analysis identified two super clusters of metagenome-assembled genomes (MAGs) and three sample clusters in our FMT cohort. MAG super-cluster I dominated the pediatric healthy controls, donor D2 and its FMT recipients, whereas donor D3 and its recipients were dominated by MAG super-cluster II (Fig. 3C), an observation that was also apparent by ASV-based beta-diversity metrics (Fig. 3A). Overall, our results demonstrate donor-specific microbiome engraftment in FMT recipients by multiple methods, including the use of metagenomic assembly as a bioinformatics approach that has not been used in pediatric FMT recipients.

### Donor-specific ASV engraftment is associated with clinical FMT response

**Fig. 4:**
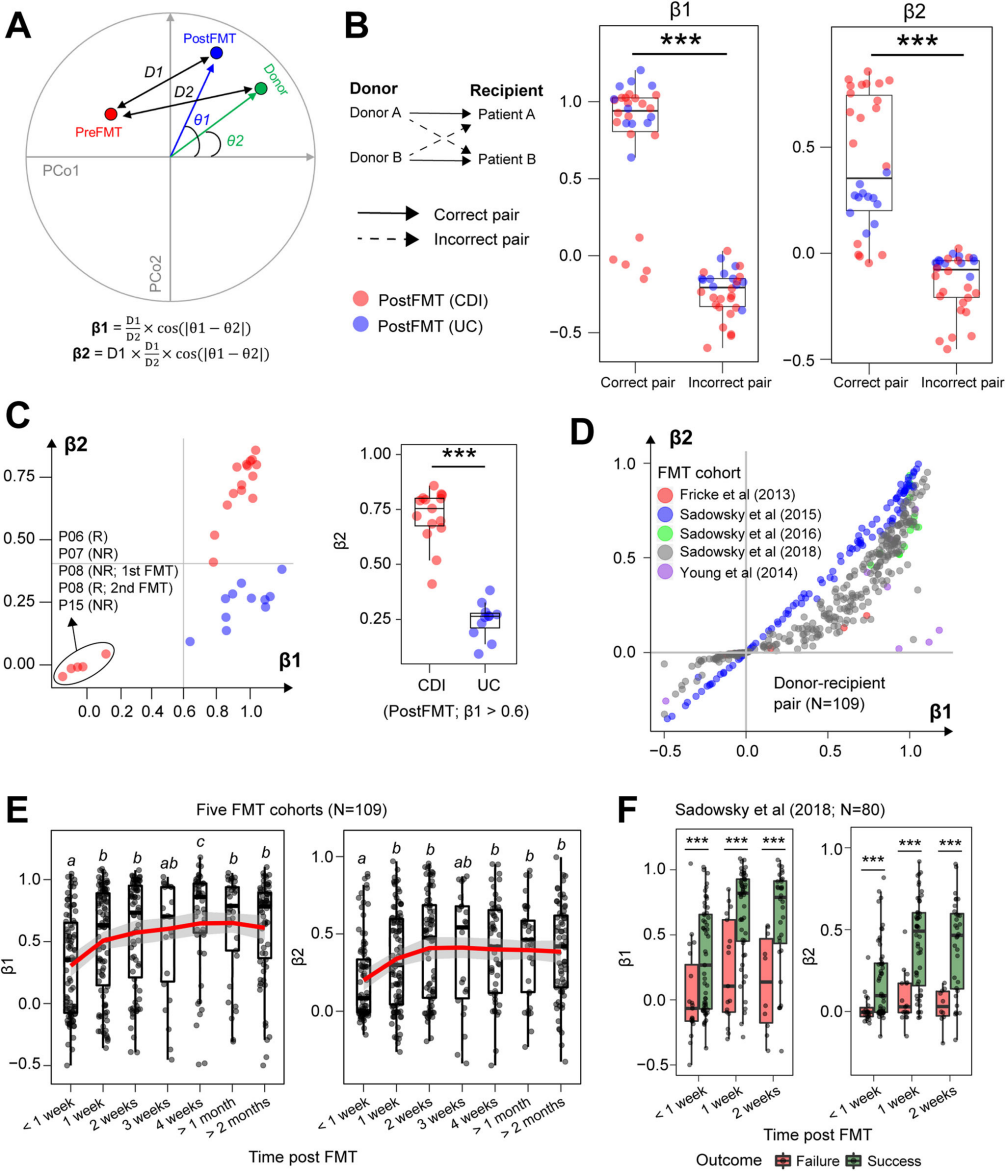
Modeling donor-specific microbiota engraftment in multiple FMT studies. **A** Mathematical formulae for calculating relative (*β*1) and absolute (*β*2) engraftment indices post-FMT using donor-recipient pairings. **B** Matched donor-recipient pairings have significantly higher engraftment index scores. Wilcoxon test: ***p < 0.001. **C** Engraftment index shows significantly higher scores in rCDI compared with UC patients receiving the same donor D2 stool. Wilcoxon test: ***p < 0.001. **D** Engraftment indices calculated for five rCDI FMT cohorts based on donor-patient pairings. **E** Engraftment indices increase significantly during longitudinal follow-up of rCDI patients post-FMT. Pairwise Wilcoxon test with Benjamini–Hochberg correction: italic script (*a-b*) with no letters in common indicates *p* < 0.05 between groups. **F** Engraftment indices significantly differentiate responders from non-responders within the first two weeks post-FMT. Wilcoxon test: ***p < 0.001.

To assess whether donor-specific microbiota engraftment correlates with FMT outcome we generated two new engraftment indices based on spatial distancing of ASV-based beta-diversity metrics. Engraftment indices *β*1 and *β*2 were calculated to qualitatively and quantitatively measure donor ASV engraftment, respectively (Fig. 4A; see description in “Methods” section). To establish specificity of our engraftment indices we measured different donor-recipient pairings in our pediatric FMT cohort. We found that matched donor-recipient pairings improved engraftment indices significantly over mismatched pairings (Fig. 4B). This finding implies the potential practicality of our engraftment tracking model in accurately identifying specific FMT donors. Furthermore, we corroborated our engraftment index results through the application of SourceTracker2, a supplementary tool, which consistently verified the corresponding donor for each patient with a commendable level of microbiome restoration (Supplementary Data).

To gain insight into the impact of PreFMT microbiome complexity on donor-specific engraftment, we conducted a comparative analysis of microbiome engraftment in patients with rCDI and ulcerative colitis (UC) who received universal donor materials but underwent different PreFMT treatments. Our findings revealed no statistically significant differences in *β*1 values between UC and rCDI patients. However, we observed significantly lower *β*2 values in PostFMT samples of UC patients compared to those of rCDI patients, with *β*1 values exceeding 0.6 (Fig. 4C). These results are consistent with the beta-diversity distances observed between PreFMT and PostFMT samples of UC and rCDI patients (Fig. 3A). As reported previously^13^, donor microbiota engraftment was much less efficient in ulcerative colitis patients who did not receive vancomycin pretreatment before FMT (Fig. 4C). Thus, our engraftment index *β*1 serves as a qualitative indicator, characterizing the extent of engraftment of donor ASVs/strains within the recipient population. On the other hand, *β*2 represents a quantitative metric, providing a measure of the specific engraftment attributed to the donor.

Although the sample size of our pediatric study is not powered for rigorous statistical analysis, we found that clinical FMT success was associated with an engraftment index score of *β*1 > 0.6 or *β*2 > 0.2, whereas lower engraftment scores (*β*1 < 0.2) identified with FMT failures (Fig. 4C). This finding was consistent across the entire pediatric FMT cohort demonstrating that adult donor stool efficiently engrafts in children aged between 2 and 18 years (Supplementary Fig. 7). To assess the general utility of our engraftment indices in predicting clinical FMT responses, we calculated donor-specific ASV engraftment in five independent rCDI cohorts where matched donor-recipient pairing information was reported^20–24^ (Fig. 4D). Using longitudinal ASV amplicon data generated from 109 rCDI patients undergoing FMT, we demonstrated statistically significant donor-engraftment (*β*1 and *β*2) within 1 week of treatment and stable engraftment was evident throughout 8 weeks representing the primary clinical end point (Fig. 4E). By stratifying patients based on clinical FMT response, we validated our engraftment index thresholds of *β*1 > 0.6 or *β*2 > 0.2 as being predictive of clinical efficacy after 2 weeks of treatment (Fig. 4C, F).

### Clinical response is reflective of probiotic strain engraftment in functional gastrointestinal disorders

**Fig. 5:**
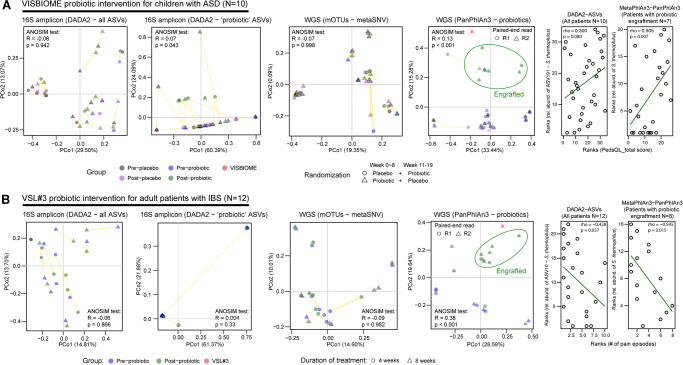
Strain tracking of probiotics improves the interpretation of clinical outcomes. **A** VISBIOME probiotic intervention of children with autism spectrum disorder (ASD). **B** VSL#3 probiotic intervention of adults with irritable bowel syndrome (IBS). ‘Probiotic’ ASVs are selected based on a specific species match contained in the probiotic product (BLASTn hits with 100% identity and 100% coverage). Abundance-weighted Jaccard distance metric was used for beta-diversity analysis of ASV profiles, while binary Jaccard distance metric was used for mOTUs-metaSNV allele frequency and PanPhlAn3 gene composition profiling. Clinical outcomes (PedsQL score and abdominal pain episodes) were correlated with probiotic engraftment measured by ASV versus PanPhlAn3.

While we successfully applied DADA2-ASV and metaSNV-based variant calling to demonstrate clinical efficacy with donor-specific microbiome engraftment in rCDI patients, it remains unclear whether similar methods can be applied to predict clinical efficacy more broadly to encompass defined microbiota products or probiotics. To test this possibility, we performed a metagenomic interrogation of gastrointestinal symptoms improvement scores with probiotic strain engraftment in two interventional cohorts that we have previously reported^25,26^. These included adults with irritable bowel syndrome (IBS) and children with autism spectrum disorder (ASD), suffering from gastrointestinal symptoms. These patients received a daily oral VSL#3 or VISBIOME dose up to 8 weeks, respectively^25,26^. Contrary to our FMT findings, DADA2-ASV and mOTUs-metaSNV profiling failed to differentiate pre- from post-probiotic fecal samples in either of our interventional cohorts indicating a limitation in tracking the engraftment of defined bacterial strains that are indistinguishable at the 16S level or using the 10 universal marker gene set provided in the mOTUs database (Fig. 5A, B and Supplementary Data). Distinguishing pre- from post-treatment specimens improved marginally when we focused the beta-diversity analysis on probiotic-targeted ASVs containing annotations of the same species comprising the probiotic products. However, we only detected significant separation in the pediatric ASD cohort (generated with Illumina-16S data) and not the adult IBS cohort (generated with 454-16S data), a difference that could be explained by the variation in sequencing depth and/or protocol differences (Fig. 5A). Therefore, we utilized PanPhIAn3, a strain-level metagenomic profiling tool for identifying the gene composition of individual strains, to detect the engraftment of specific probiotic strains in patients. By combining gene composition of all bacterial species detected from the metagenomic data of probiotic products and patient samples, we achieved significant separation between pre- and post-probiotic samples through beta-diversity analysis (Fig. 5A, B). The major distinguishing feature was the engraftment of *Streptococcus thermophilus* from the two probiotic products in the majority of follow-up samples (Supplementary Fig. 8 and Supplementary Data). The precise detection of probiotic strains allowed us to correlate clinical symptoms with engraftment (Fig. 5A, B), which was significantly improved for measures of abdominal pain and quality of life (PedsQL GI) scores with *Streptococcus thermophilus* abundance. DADA2-ASV and metaSNV profiling is therefore not able to accurately assess engraftment of probiotics or defined bacterial consortia, but this limitation can be overcome by using other strain-level profiling tools.

### Microbiota engraftment is coupled to functional restoration but fails to demonstrate donor-specific metabolite signatures

**Fig. 6:**
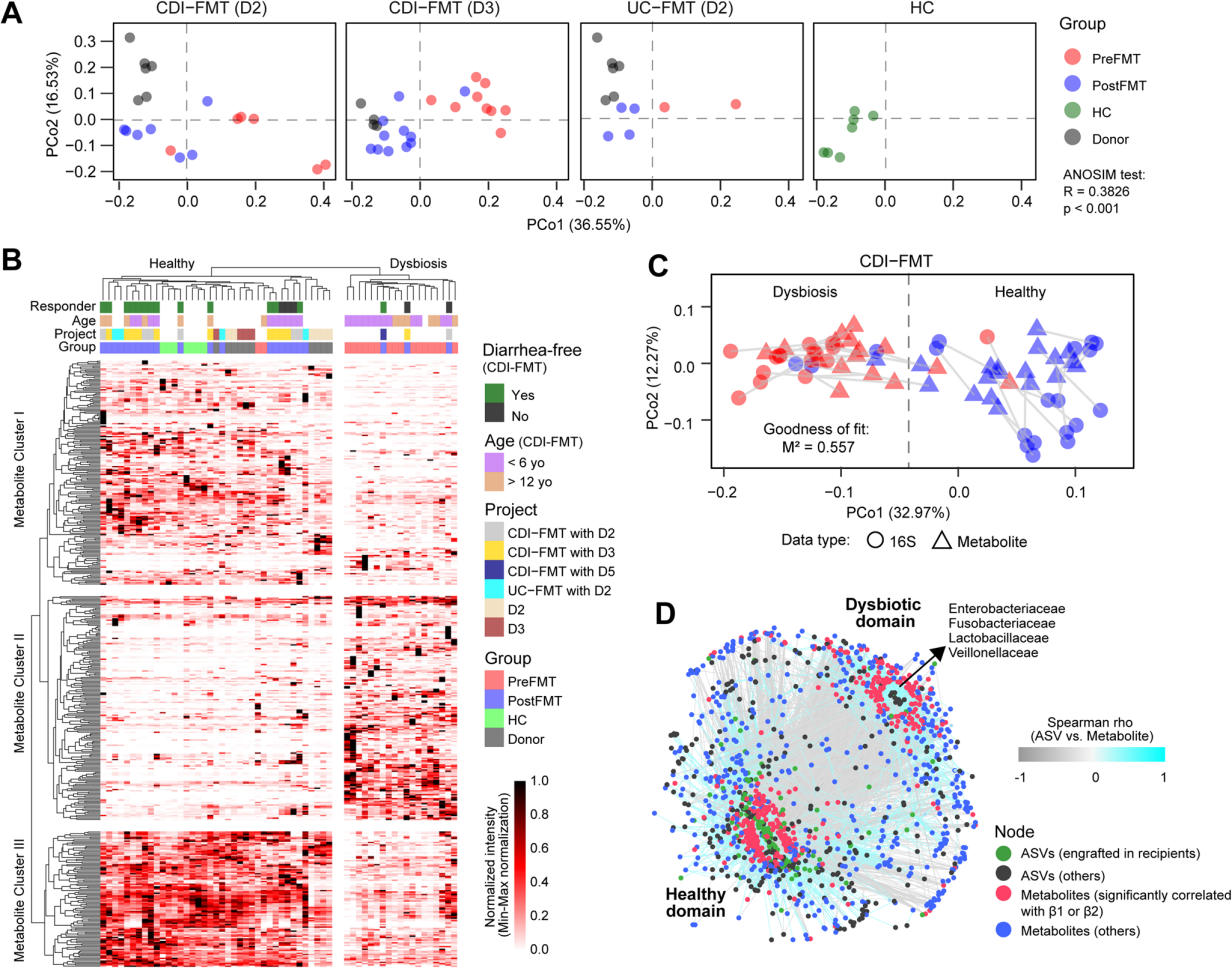
Fecal metabolomics shows metabolite reconstitution post-FMT but fails to demonstrate donor-specific restoration. **A** Principal coordinate analysis of fecal metabolites differentiates pre- from post-FMT samples. **B** Hierarchical clustering analysis identifies three metabolite clusters that differentiate healthy from dysbiotic subjects. Metabolites that showed significant (FDR < 0.05) spearman correlation with engraftment index *β*1 or *β*2 were only included in the analysis. **C** Procrustes analysis shows high concordance between metagenomic and metabolomic profiles that differentiate pre- from post-FMT specimens. Abundance-weighted Jaccard distance metric was used for distance calculation. **D** ASV-metabolite bipartite network analysis differentiates healthy microbiome from dysbiotic communities. ASVs and metabolites with significant (FDR < 0.05) Spearman correlations and coefficients >0.4 or <−0.4 were only included in the network analysis.

We demonstrated that donor-specific microbiota engraftment occurs, but it remains unclear whether donor variants drive unique functional signatures in FMT recipients. To address this question we performed unbiased global metabolomics and compared the differential abundance of fecal metabolites in matched FMT donors and recipients. Using unconstrained ordination and hierarchical clustering analyses of stool metabolomics data, we identified significant metabolite superclusters that differentiated pre- and post-FMT specimens but failed to show donor-specific functional restoration (Fig. 6A, B). These findings demonstrate the metabolic redundancy of donor species and strain variants engrafted in FMT recipients^27^. By assigning specific metabolite clusters to clinical FMT outcomes, we demonstrated higher dipeptide diversity in metabolite cluster III that was significantly associated with successful FMT and healthy age-matched individuals (Supplementary Fig. 9). Profiling of stool dipeptide diversity is therefore of potential utility as a functional biomarker of the healthy host microbiota interactome reflecting diverse proteolytic and/or peptidylic activities. This finding was validated through omics integration of microbiome and metabolome signatures by Procrustes and bipartite network analysis, which identified distinct patient outcome clusters associated with FMT efficacy (Fig. 6C, D).

### Age-dependent metabolite restoration after FMT

**Fig. 7:**
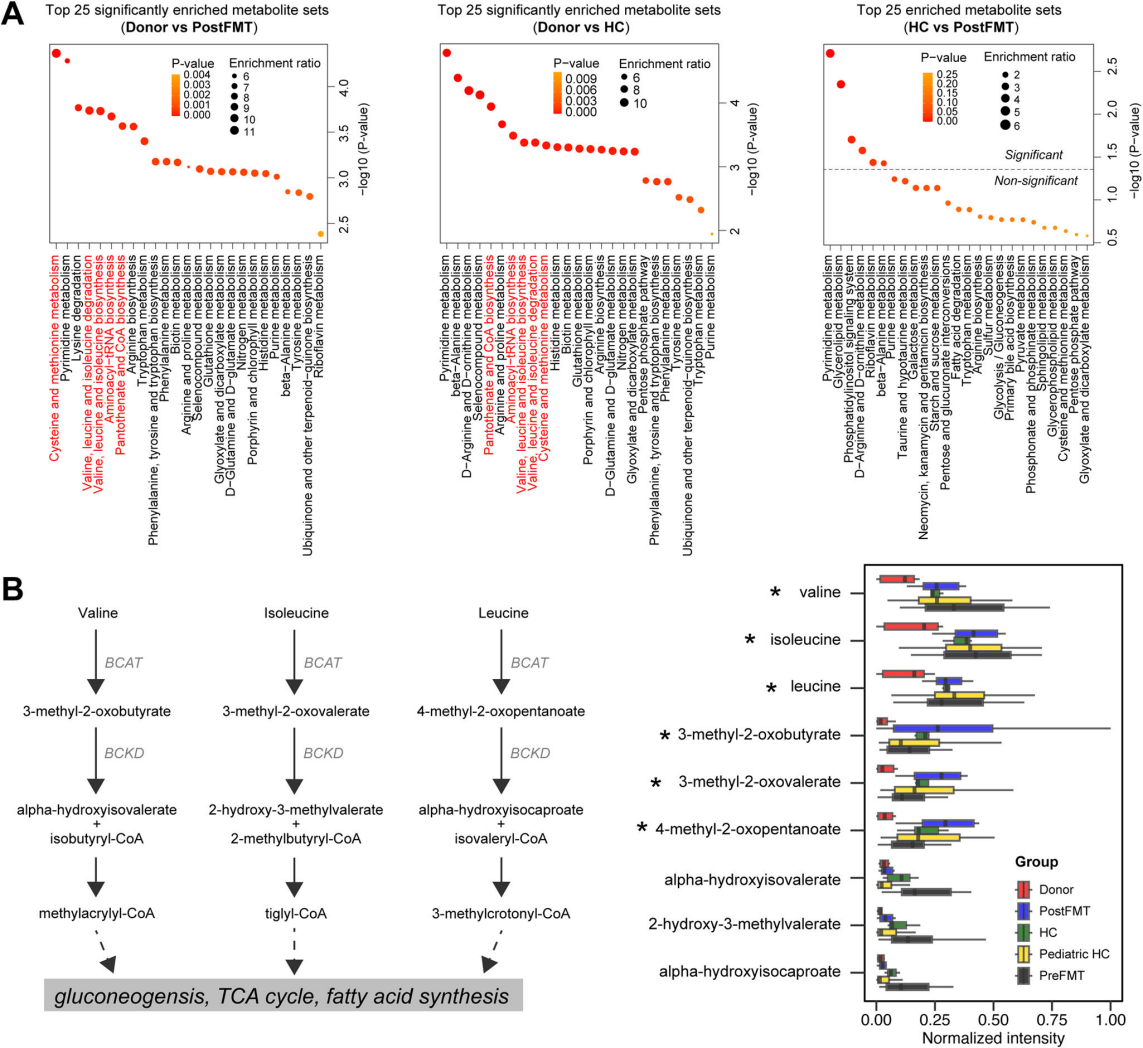
Metabolomics identifies pediatric-specific pathways that restore independently of adult donor engraftment. **A** Metabolite set enrichment analysis identifies significant metabolic pathways that are conserved in healthy children (HC and PostFMT). Top 5 pathways that differentiate healthy pediatric from adult donors are highlighted (red). **B** Branched-chain amino acid degradation is significantly elevated in post-FMT and healthy children. Kruskal-Wallis test with BH procedure for donor, PostFMT (*β*1 > 0.6) and HC samples: *p < 0.05. Denotations: BCAT branched-chain aminotransferase, BCKD branched chain ketoacid dehydrogenase.

To ascertain whether there is age-appropriate functional restoration following stable engraftment (*β*1 > 0.6) of adult donor microbiome features in pediatric patients with rCDI, we conducted a comparative analysis of metabolomics profiles in adult donor stool with pre- and post-FMT specimens. Our findings revealed 73 differentially abundant metabolites in healthy pediatric FMT recipients compared to adult donor stool (Supplementary Fig. 10). These differential metabolites underwent further metabolic enrichment analysis, which identified five age-dependent metabolic functions, including cysteine and methionine metabolism, aminoacyl-tRNA biosynthesis, pantothenate and CoA biosynthesis, and valine, leucine and isoleucine biosynthesis and degradation in FMT recipients (Fig. 7A and Supplementary Data). The enrichment of these metabolic pathways was further confirmed in healthy age-matched children, indicating that pediatric-specific metabolite restoration occurs independently of adult microbiome-derived signals (Fig. 7A). However, no significant differences were observed for these pathways from non-functional metagenomic profiling (Supplementary Fig. 11A), suggesting a strong discordance between metabolic and metagenomic profiling. Our metabolic profiling also revealed increased activity of microbial branched-chain aminotransferase (BCAT) in response to pediatric-enhanced degradation of the branched-chain amino acids (BCAA), including valine, leucine and isoleucine, which was not detected by metagenomic profiling (Supplementary Fig. 11B). This observation was validated in another independent healthy pediatric cohort (Fig. 7B).

## Discussion

Microbiota engraftment is a well-recognized parameter for assessing the effectiveness of FMT in patients with rCDI or inflammatory bowel disease, both being disorders associated with gut microbiota imbalance^28,29^. However, previously reported engraftment indices using alpha-diversity, dissimilarity calculation, or taxonomic abundance did not precisely identify donor-specific microbiome engraftment^8–10^. These methods are not designed for strain-level inference due to the limitation of upstream processing, i.e., 97% similarity used for 16S amplicon sequence clustering^30^. While these methods were successful in predicting overall microbiome community restoration and even clinical outcome in rCDI recipients post-FMT, they are not able to establish a precise link between the donor microbiome and the recipient’s engraftment pattern, which requires strain-level profiling. In the meantime, metagenomics-based strain tracking can also be problematic as it relies on metagenomic variant profiling that requires deep sequencing and computationally intensive processing^11–14^.

An alternative approach for strain-level inference is to use sequence denoising for 16S amplicon data, which has been shown to differentiate strains based on single nucleotide differences in ASVs^15^. Using mathematical modeling, we successfully showed specific donor ASV engraftment in FMT recipients. We further developed a novel engraftment index that correlated significantly with clinical efficacy and validated this approach against multiple FMT cohorts. Because our model could predict clinical outcomes based on donor ASV engraftment, this reference-independent, taxonomy-agnostic approach has potential in clinical utility for monitoring FMT outcomes in a rapid and computationally cost-effective manner.

Although the ASV approach identifies strains of some species, it is limited by the amplicon length and single marker gene used. We highlight this limitation of the ASV approach in confidently detecting strains from defined bacterial consortia or probiotic products in recipients, which could be more precisely identified with more advanced strain-level profiling tools using deeply sequenced metagenomic data. The inherent problems of identifying and quantifying probiotic products in patient fecal specimens are well recognized, although long-read sequencing strategies that generate multiple full-length targeted amplicons could improve strain-level inference^31^. We demonstrated broad utility when adopting strain-level resolution to assess the clinical efficacy of probiotic consortia engraftment in adults and children with functional gastrointestinal disorders. The precise detection of probiotic strains through metagenomics profiling provided a more significant correlation of symptom relief with bacterial engraftment. Alternatively, this could also reflect poor compliance in consuming probiotic product by clinically non-responsive subjects.

FMT has also emerged as an effective treatment for rCDI in children. However, as microbiome development in children differs from that of adults, it remains unclear whether adult fecal donors can provide effective age-appropriate functional restoration in pediatric patients. Our analyses revealed that adult donor-specific engraftment dominated in pediatric rCDI recipients, with high concordance observed between donor-specific strain engraftment and functional metabolite restoration. Our integrated systems approach also demonstrated that concordant donor strain engraftment with metabolite restoration is associated with FMT outcomes in both adults and children with rCDI. While donor-specific strain engraftment predicted FMT outcomes, we observed donor functional redundancy in metabolic restoration. Interestingly, significant age-related metabolic dependencies were evident after FMT, including elevated BCAA degradation, which has been reported to provide a fitness benefit for muscle, immune and brain development^32^. Some developmental-specific metabolic functions are therefore retained after adult fecal donors are used to treat pediatric patients, findings that should contribute to the ongoing debate regarding the appropriateness of age-matching of fecal donors in pediatric FMT.

One expected outcome of successful microbiota engraftment is the restoration of gut metabolites, such as elevated levels of butyrate and secondary bile acids post-FMT^21,33^. While engraftment of adult microbiota in pediatric rCDI patients was observed, another strength of the present study was to provide a unique metabolome comparison between adult donors and pediatric recipients, such as BCAA metabolism, which was identified as a pediatric-specific trait. Although *C. difficile* could utilize BCAAs through Stickland reactions^34^, BCAAs have been shown to enhance the binding of the global regulator CodY to the tcdR promoter region, thereby repressing toxin gene expression in *C. difficile*^35^. BCAAs metabolism has been implicated in the differentiation of active infection versus asymptomatic carriage of *C. difficile*^36^ and might provide an alternative explanation for the high prevalence of *C. difficile* in young asymptomatic children^37^. Additionally, specific gut bacteria use BCAAs as precursors in the production of branched short-chain fatty acids (BSCFAs) that increase IgA-related immune homeostasis^38^. Overall, our results highlight the presence of pediatric-specific metabolite signatures regardless of gut microbiome engraftment.

## Methods

### Patient cohorts

FMT used in the treatment of pediatric rCDI and ulcerative colitis patients was conducted from 2013 to 2016 at Texas Children’s Hospital under IRB-approved informed consent (#H-31066) at Baylor College of Medicine. Preliminary 16 S rDNA amplicon data-based microbiome findings were recently reported^17^. Deposited sequencing data from adult FMT cases was used for study validation^20–24^. Two interventional studies previously reported by us for probiotic VSL#3 or VISBIOME treatment in adult IBS and pediatric autism spectrum disorder were used for validating strain tracking^25,26^.

### Stool microbiome sequencing

Stool samples collected from patients and FMT donors were subjected to DNA extraction using the PowerSoil^®^ DNA Isolation Kit (Qiagen, Hilden, Germany) following manufacturer’s instructions. Shotgun metagenomic sequencing was performed by BGI Genomics Co., Ltd. (Hong Kong, China) requiring a minimum DNA input of 200 ng. Sample QC, shotgun library construction and sequencing were all carried out by the BGI service team generating 2 × 150 bp paired-end reads.

### 16S amplicon data analysis and meta-analysis of FMT datasets

For 16S amplicon data analysis, raw paired-end reads were merged with vsearch (version 2.9.0) using default parameters. DADA2 (version 1.8.0)^15^ was then used to perform quality filtering (maxEE=2) and sequence denoising to generate amplicon sequence variants (ASVs) profiles following its recommended procedures. Taxonomic annotation for ASV sequences was performed with the IdTaxa function in the R package DECIPHER (version 2.6.0)^39^ and its pre-built 16S training dataset (SILVA release132). BLASTn search was run for specific ASVs against the NCBI 16S rRNA reference database and top hits with 100% coverage and 100% identity were used for potential species annotation. The AlignSeqs function in the DECIPHER package was used to perform multiple sequence alignment for ASVs, and an approximately-maximum-likelihood phylogenetic tree was built with FastTree (version 2.1.3) for the sequence alignment. For analyzing other publicly available 16S datasets, sequence denoising analysis was performed as indicated above. Total sum scaling method was used to normalize ASV profiles without random rarefaction process. Due to the substantial reduction in singleton occurrences achieved through the denoising approach and the lack of reliable ground truth data for real-world microbiome datasets, we opted not to employ ASV abundance filtering or ASV prevalence filtering in this study. These methods have the potential to eliminate distinctive features that differentiate donors, which was the primary focus of our investigation. However, for benchmarking purposes exploring the contribution of potentially spurious ASVs (not chimeras) in donor-specific engraftment, we implemented abundance filtering utilizing the "filter_otus_from_otu_table.py" script, employing a parameter of “--min_count_fraction” set at 0.01%.

Using ASV count information, SourceTracker2^40^ was used to analyze CDI-FMT samples (donor and PreFMT samples were labeled as source, PostFMT samples were labeled as sink). Unconstrained ordination analysis using principal coordinate analysis (PCoA) or non-metric multidimensional scaling (NMDS) was performed with different distance metrics including binary Jaccard distance, abundance-weighted Jaccard distance, weighted and unweighted UniFrac distance, and Bray–Curtis dissimilarity index calculated for ASV relative abundance profile. Analysis of Similarities (ANOSIM) test was performed with the anosim function in the vegan package for the distance profile to statistically measure significant differences between two or more sample groups using a permutation of 999. Fitting factors (i.e. ASVs) onto a two-dimensional ordination plot (first two coordinates) was achieved by using the envfit function in the vegan package (version 2.6-2). Significance of fitted factors was established using the permutation of 999 in the envfit run. Patient-ASV bipartite networks were generated in the Cytoscape^41^ tool by using the Edge-weighted Spring Embedded Layout algorithm.

### Shotgun metagenomic data analysis

Quality trimming of raw paired-end reads was performed by vsearch (version 2.9.0) with the maxEE of 1 and a minimum length of 100 bases, and further re-pairing with bbmap (version 38.34). Three read mapping based taxonomic profiling tools – MetaPhlAn3 (version 3.0.7)^42^ with its pre-built database (mpa_v30_CHOCOPhlAn_201901), mOTUs (version 2.6)^43^ with its default database, and Kraken2 (version 2.08)^44^ with its pre-built database (minikraken2_v2_8GB_201904_UPDATE) were then applied to analyze shotgun metagenomes using their default parameters. HUMAnN3 (version 3.6) was used for profiling metabolic pathways and gene families using MetaCyc and KEGG databases^42^.

Metagenomic variant calling pipeline metaSNV (version 1.0.3) implemented in mOTUs (version 2.6)^43^ was used to generate variant calling profiles with the custom parameters (-fb 0.01 -fd 0.001 -fm 1 -fc 0.001) providing sensitive detection of substitutions. Filtered allele frequency tables were then treated as input matrix for calculating binary Jaccard distance followed by PCoA analysis. Another strain detection analysis was performed with PanPhlAn3 by mapping sequence reads to the pre-built pan-genome database of specific species, followed by a profiling step using custom parameters (--min_coverage 1 --left_max 1.70 --right_min 0.30). Read counts of pan-genome gene tables were then treated as input matrix for calculating binary Jaccard distance followed by PCoA analysis. ANOSIM test was performed with the anosim function in the vegan package for the binary Jaccard distance profile to statistically assess group difference using a permutation of 999. The third metagenomic strain tracking tool – SameStr (version 1.2023.04-1)^18^ using the MetaPhlAn3 database was adopted using default parameters.

Metagenomic de novo co-assembly analysis was performed as described below: human sequence reads were filtered by using bowtie2 (version 2.3.4.3) and human reference genome (version hg19), followed by digital normalization using the script bbnorm.sh in bbmap (version 38.34) with custom parameters (target=40 min=5). Normalized reads from all samples were concatenated for de novo co-assembly by megahit (version 1.1.4) with custom parameters (--min-contig-len 2000). Metagenomic assemblies were used as the input for downstream analysis using the Anvi’o platform (version 7)^19^ following the instructions provided for regular metagenomic analysis workflow. Specifically, we selected metabat2 implemented in the anvi-cluster-contigs command for metagenomic binning of this large dataset, and guided refinement was further performed for one metagenomic bin with high redundancy (>10%). Gene calls was achieved with the anvi-get-sequences-for-gene-calls command, whereas functional annotation was performed with EggNOG-mapper (version 2.0.1). The anvi-estimate-scg-taxonomy command was used to infer the taxonomy for metagenomic bins (redundancy<10%) after identifying single-copy core genes. The anvi-summarize command was used to generated output profiles, and Anvi’o view – detection profile (proportion of nucleotides in a contig that are covered at least 1X) was used for downstream hierarchical clustering analysis using Euclidean distance and Ward clustering algorithms.

### Generation of FMT engraftment indices

Donor-recipient pairing information is an essential requirement for calculating engraftment indices, which compare matched pre- and post-FMT with donor fecal samples. Our approach involves utilizing beta-diversity analysis of ASV profiling to determine donor-specific microbiota engraftment in FMT recipients. We utilize PCo1 and PCo2 values obtained from the beta-diversity analysis to compute distances D1 between pre- and post-FMT samples, and D2 between donor and post-FMT coordinates. The degrees *θ*1 and *θ*2, calculated from post-FMT and donor coordinates respectively, are utilized to generate two engraftment indices:

$${\upbeta}1 = \frac{{{{{\mathrm{D}}}}1}}{{{{{\mathrm{D}}}}2}} \times {{{\mathrm{cos}}}}\left( {\left| {{\uptheta}1 - {\uptheta}2} \right|} \right)\;{{{\mathrm{and}}}}\;{\upbeta}2 = {{{\mathrm{D}}}}1 \times \frac{{{{{\mathrm{D}}}}1}}{{{{{\mathrm{D}}}}2}} \times {{{\mathrm{cos}}}}\left( {\left| {{\uptheta}1 - {\uptheta}2} \right|} \right)$$

The *β*1 value provides a qualitative measure of donor-specific engraftment, where a value of 1.0 indicates 100% engraftment of FMT recipient by the donor microbiota. The *β*2 value, on the other hand, represents a quantitative measure of donor-specific microbiota engraftment, while also describing microbiota differences between pre- and post-FMT specimens. A higher *β*2 value signifies more significant microbiome engraftment and reflects higher recipient receptiveness to donor microbiome engraftment.

### Stool metabolomics

Lyophilized fecal specimens were forwarded to Metabolon Inc. to perform global HD4 metabolite profiling. Abundance-weighted Jaccard distances were calculated for metabolomics signatures, followed by principal coordinate analysis. The microbiome (ASVs) and metabolite data were processed using common beta-diversity metrics and ordination methods, and Procrustes analysis was used to evaluate concordant configurations (shapes) of independent omics datasets. Hierarchical clustering analysis was performed using the pheatmap package (version 1.0.12; default parameters). Statistical analysis was conducted on the metabolomics profiles to assess group differences without data normalization or transformation. Metabolite set enrichment analysis was performed using MetaboAnalyst (version 5.0), referencing compounds listed in the Human Metabolome Database (HMDB). The Kyoto Encyclopedia of Genes and Genomes (KEGG) pathway database was utilized as the metabolite set library for the pathway enrichment analysis.

### Statistical analysis

Wilcoxon and Kruskal–Wallis tests were applied for group comparisons. To adjust for multiple comparisons, the Benjamini–Hochberg false discovery test was set at a threshold of 0.05. Spearman’s rank correlation coefficient was calculated using R base function cor.test(). Boxplots were generated using the ggplot2 package: data represented in the boxplots is presented as median value with the interquartile range from 25% to 75%.

### Reporting summary

Further information on research design is available in the Nature Research Reporting Summary linked to this article.

### Supplementary information

Reporting Summary

Supplementary Information

Related Manuscript File

Related Manuscript File

Related Manuscript File

## Data Availability

The 16S amplicon sequence data of our FMT cohort (accession number PRJNA735699), five public FMT cohorts (PRJNA238042, PRJNA221789, PRJNA303184, PRJEB19996, and PRJNA311224), and our two probiotic intervention cohorts (PRJNA941891 and PRJNA941893) were deposited in the NCBI Sequence Read Archive (SRA) database. Deep shotgun metagenomic sequence data of our FMT cohort (PRJNA765331), two public FMT cohorts (PRJEB23524 and PRJNA678737), and our two probiotic intervention cohorts (PRJNA725223 and PRJNA940472) were also deposited in the NCBI SRA database.

## Code availability

Code and scripts for variant calling analysis for 16S amplicon data, generating engraftment indices and performing metagenomic SNP analysis and de novo metagenomic co-assembly analysis are available at https://github.com/qinglong89/FMT_engraftment.
