# Supplementary material for: Donor-recipient specificity and age-dependency in fecal microbiota therapy and probiotic resolution of gastrointestinal symptoms
Source: NPJ Biofilms Microbiomes. 2023 Aug 3;9:54. doi: 10.1038/s41522-023-00421-4 (PMC10400536; doi:10.1038/s41522-023-00421-4)
Supplement: Supplementary file 2 — Supplementary Information [file 41522_2023_421_MOESM2_ESM.pdf]

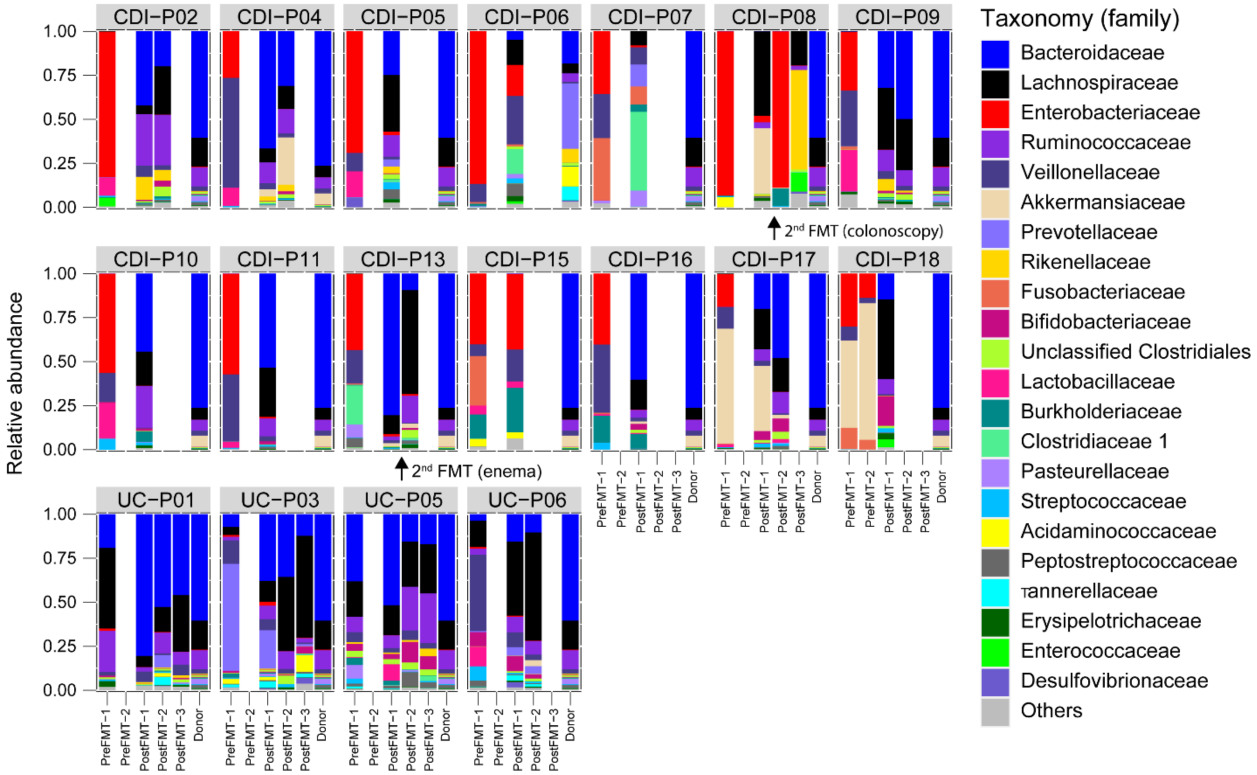

Supplementary Figure 1. Relative family abundance for each patient and paired donor. Taxonomy was collapsed down to family level from ASV tables generated by the DADA2-IdTaxa pipeline using the SILVA 132 reference database.

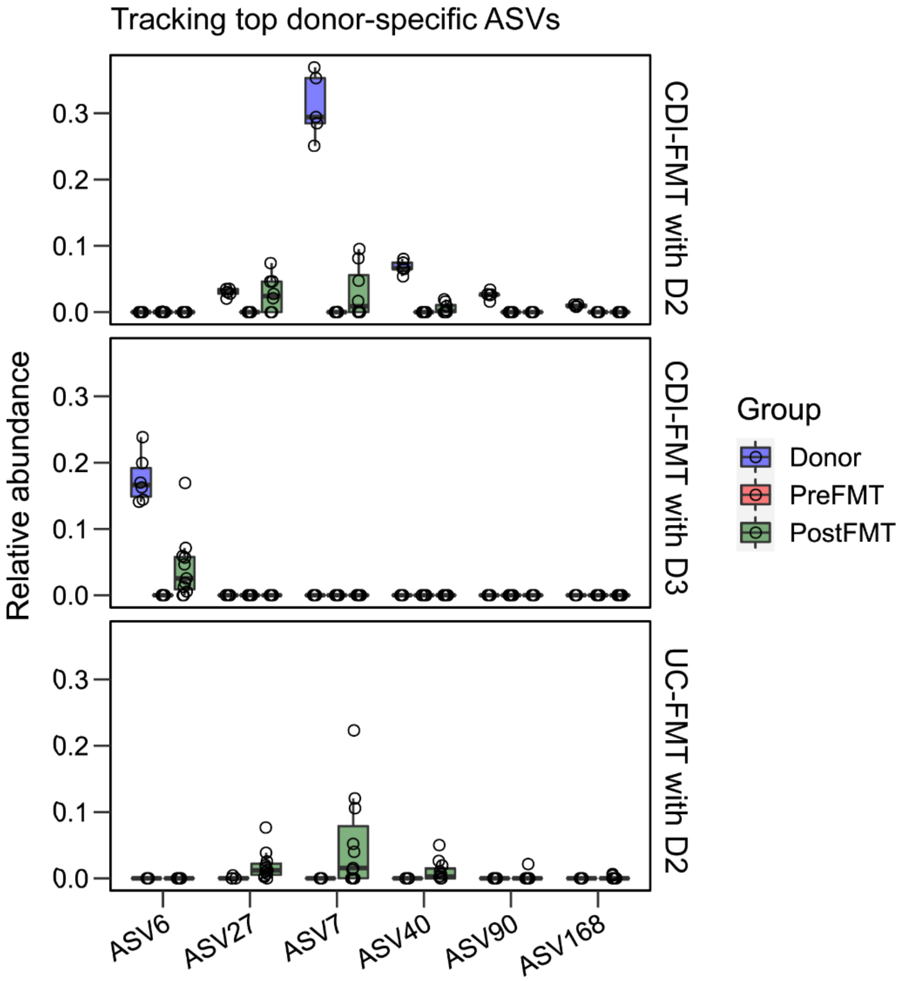

Supplementary Figure 2. Tracking top-ranked donor-specific ASVs in FMT recipients. Top donor-specific ASVs shown in Figure 2B-C were identified by the envfit function in the vegan package.

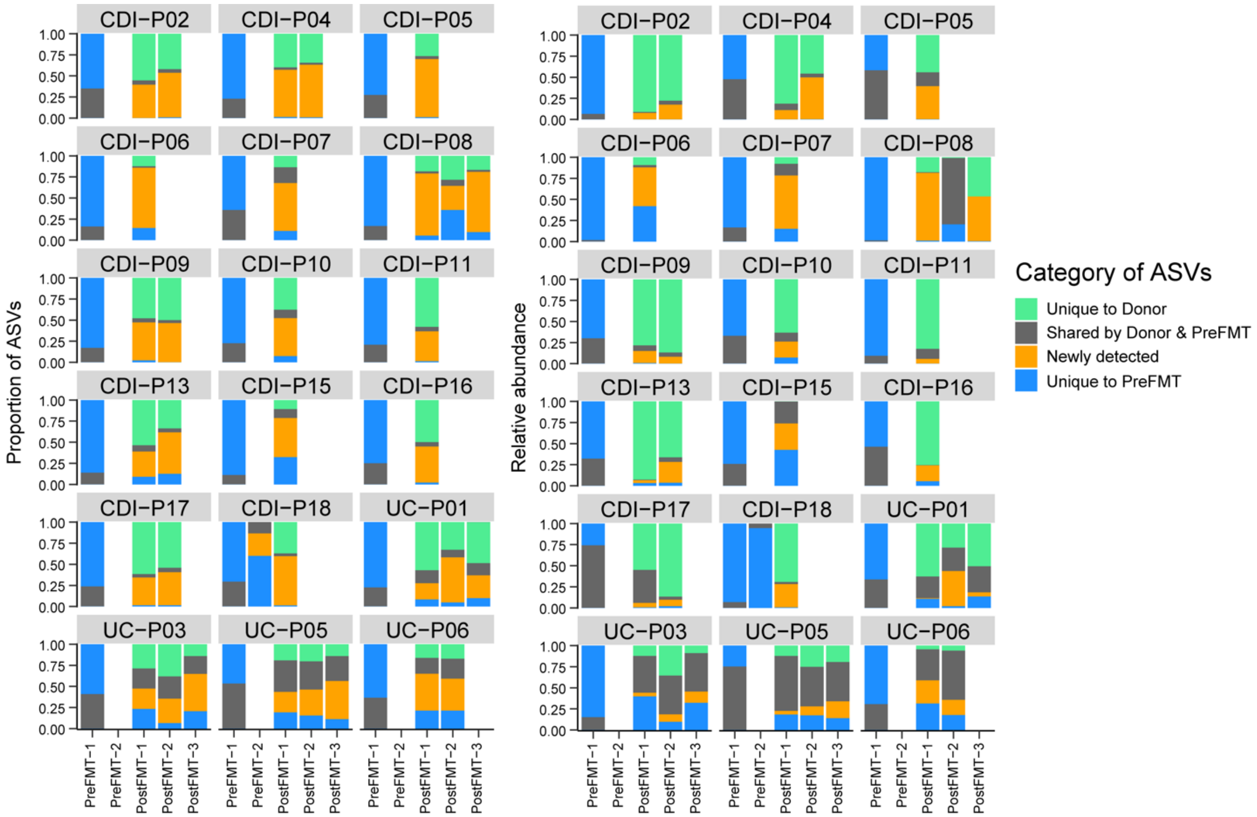

Supplementary Figure 3. **Tracking donor ASVs in FMT recipients.** Each ASV within the ASV count table was classified according to the presence or absence of data from donor, PreFMT, and PostFMT samples for each patient. These categories were as follows: 1) exclusively associated with the donor, 2) exclusively associated with PreFMT, 3) shared by both the donor and PreFMT, and 4) newly detected or engrafted. The proportion of ASVs (based on the number of ASVs) and the total relative abundance were subsequently computed for each category. These values were then graphically represented for the longitudinal samples of each patient.

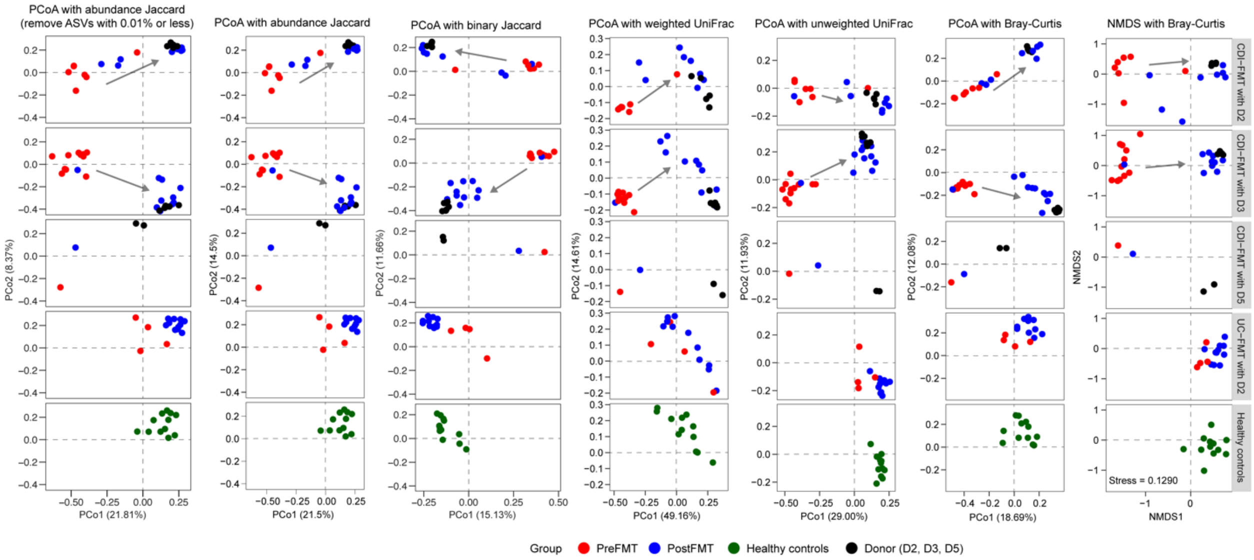

Supplementary Figure 4. Performance based analysis of distance metric and ordination method to identify donor-specific microbiota engraftment. Distance profiles were generated with four common distance metrics (binary Jaccard, abundance-weighted Jaccard, weighted and unweighted UniFrac, and Bray-Curtis dissimilarity) for ASV table without rarefaction. PCoA, principal coordinate analysis; NMDS, non-metric multidimensional scaling.

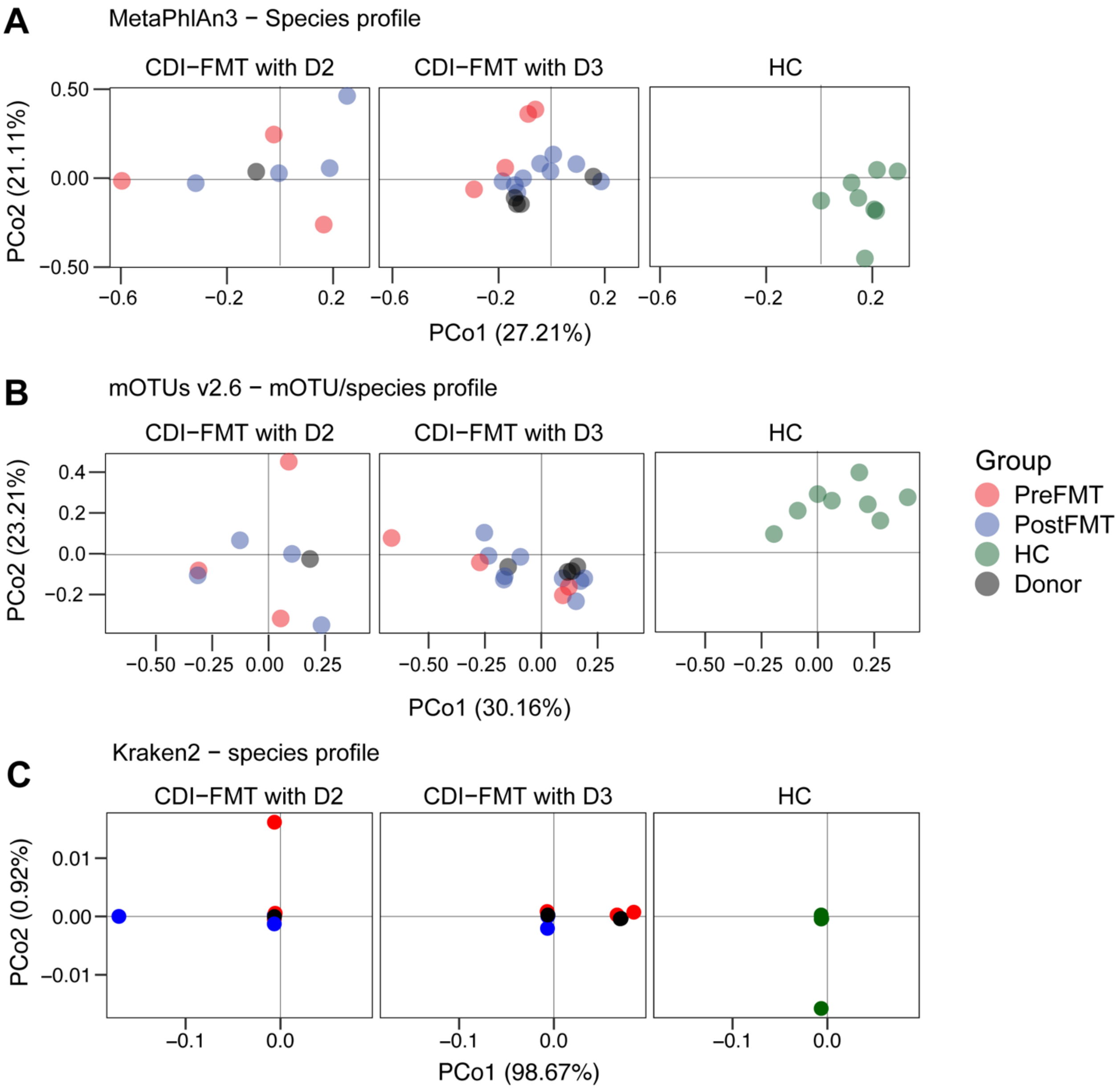

Supplementary Figure 5. **Species abundance profiling fails to demonstrate donor-specific microbiota engraftment using beta-diversity analysis.** (**A**) Beta-diversity analysis with MetaPhlan3 species abundance profile. (**B**) Beta-diversity analysis with mOTU/species abundance profile. (**C**) Beta-diversity analysis with Kraken2 species abundance profile. Beta-diversity analysis was performed with principal coordinate analysis using abundance-weighted Jaccard distance profile.

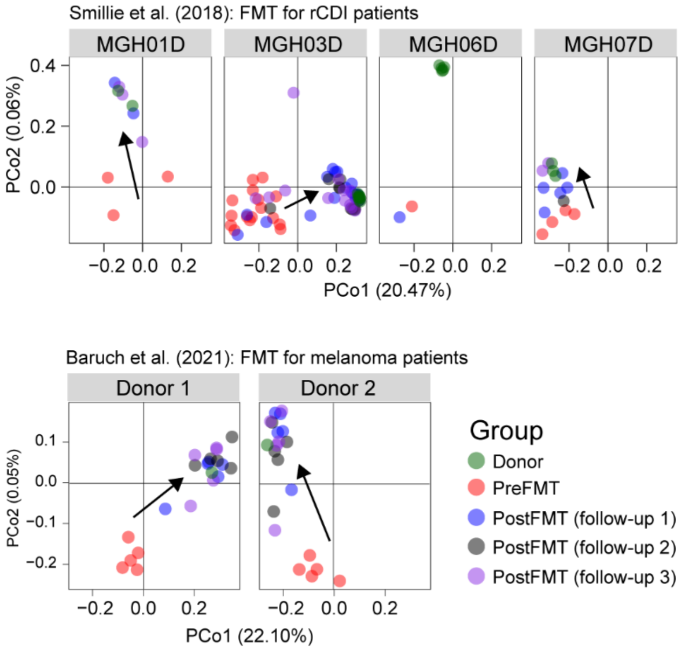

Supplementary Figure 6. Validation of donor-specific engraftment using publicly available FMT datasets with shotgun metagenomic analysis. Metagenomic SNV profiling implemented in the mOTUs pipeline was performed using two independent FMT studies of adult rCDI patients (Smillie et al. 2018) and melanoma patients (Baruch et al. 2018). SNV profiling is based on mOTUs 10 marker gene-based SNV profiling. Beta-diversity analysis was performed with principal coordinate analysis using abundance-weighted Jaccard distance profile.

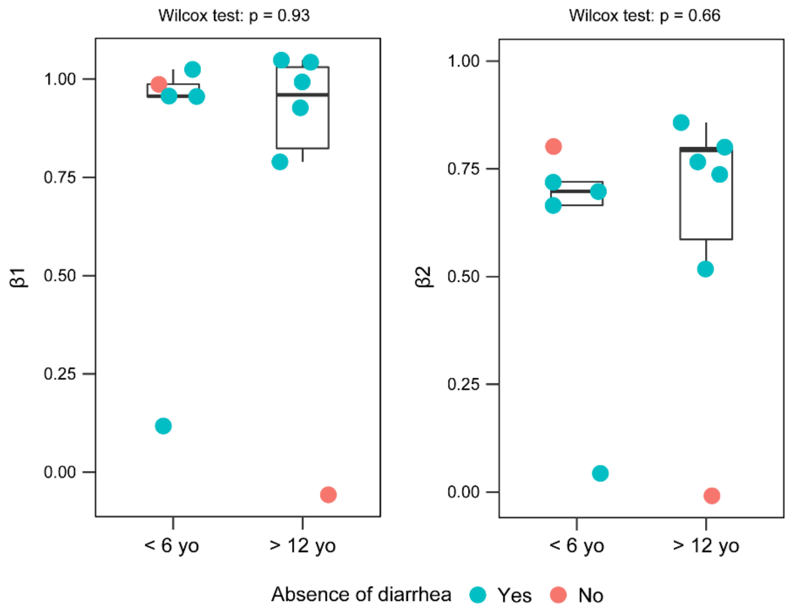

Supplementary Figure 7. Pediatric rCDI recipients with different age ranges show no significant differences in engraftment index β1 and β2. PostFMT samples at week 8 or 9 were included for statistical analysis. Absence of diarrhea is the clinical outcome measured at the primary end point (week 8 or 9).

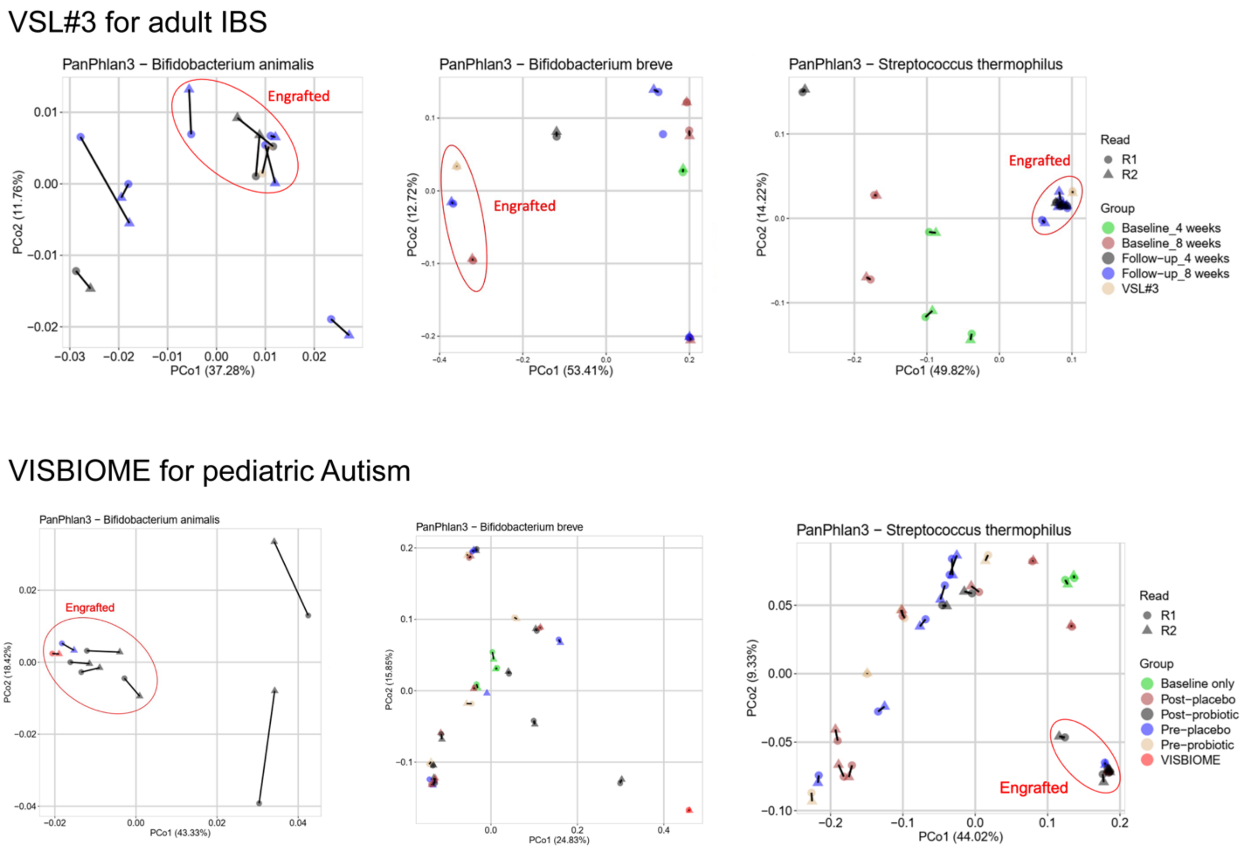

Supplementary Figure 8. PanPhlAn3 gene compositional profiling for detectable probiotic species. Beta-diversity analysis was conducted employing PCoA in conjunction with binary Jaccard distance to demonstrate the engraftment status of probiotics. For the purpose of illustration, only two species, namely *Streptococcus thermophilus* and *Bifidobacterium animalis*, were plotted due to their frequent detection in patients following probiotic intervention. WGS data were available for two probiotic products, namely VSL#3 and VISBIOME, and their respective probiotic species served as the positive controls. The proximity between the probiotic product and the post-intervention sample signifies a stronger engraftment of probiotic species within the patient.

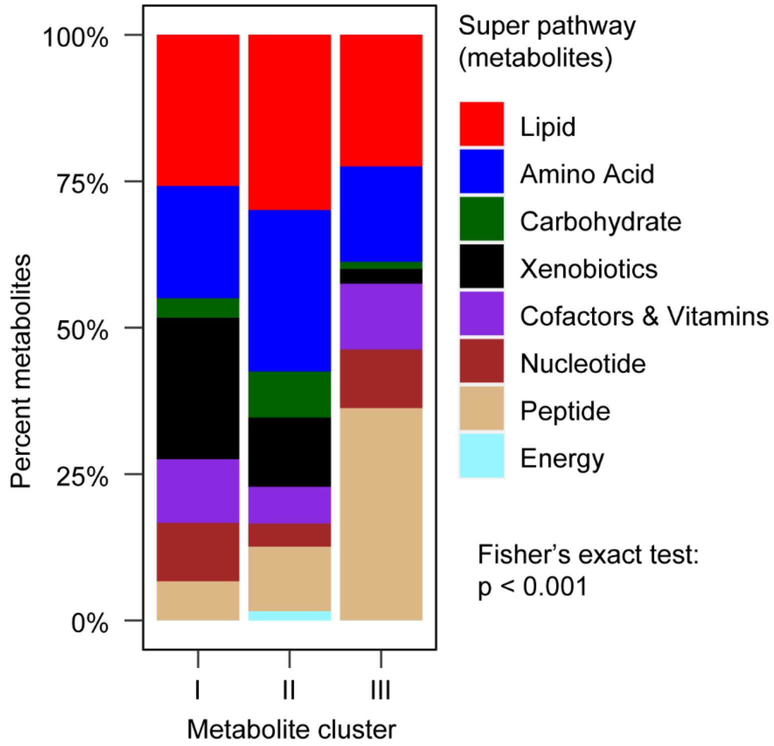

Supplementary Figure 9. Metabolite clusters are associated with specific super pathways of metabolites. Dipeptides were highly enriched and diverse in PostFMT samples and healthy subjects; majority of xenobiotics are compounds found in foods or plant derivatives as defined by Metabolon, Inc.

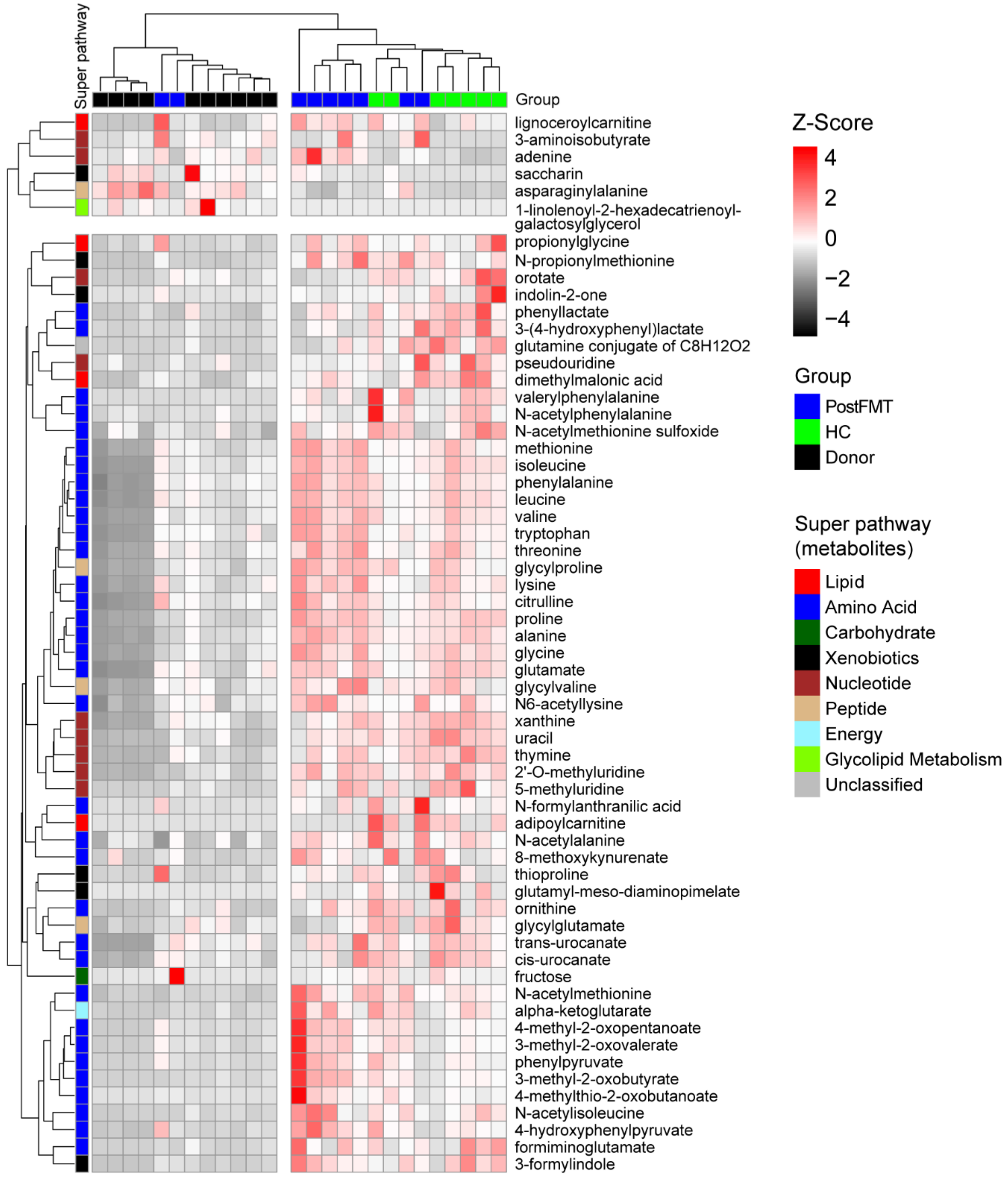

Supplementary Figure 10. Differentiating pediatric samples (HC and PostFMT) from adult donor samples by hierarchical clustering analysis of significant metabolites. PostFMT samples at week 8 or 9 with an engraftment index β1 of 0.6 or above were included for statistical analyses. Kruskal-Wallis test with BH procedure was applied for analyzing Donor, HC and PostFMT samples: *, $p < 0.05$.

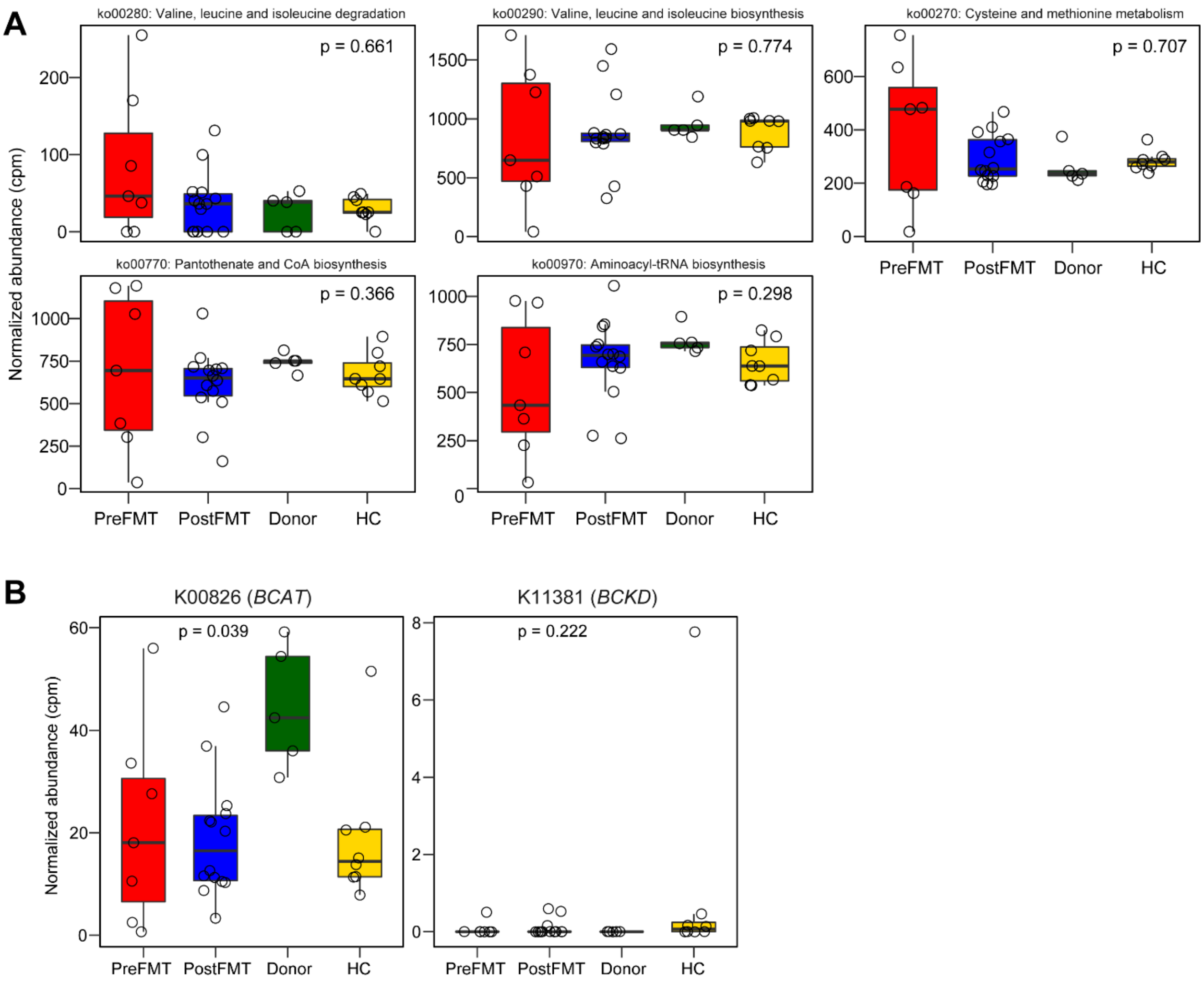

Supplementary Figure 11. Pathway and enzyme abundance predicted from metagenomic data. HUMAnN3 was used to profile pathway and gene families from shotgun metagenomic data using the KEGG database. (**A**) Normalized abundance of selected KEGG pathways. (**B**) Normalized abundance of two gene families involved in branched-chain amino acids degradation. Kruskal-Wallis test was used for group comparisons.
